# The Lives of Native Hawaiian Elders and Their Experiences With Healthcare: A Qualitative Analysis

**DOI:** 10.3389/fpubh.2022.787215

**Published:** 2022-02-22

**Authors:** Keilyn Leina‘ala Kawakami, Shelley Muneoka, Rachel L. Burrage, Leslie Tanoue, Kilohana Haitsuka, Kathryn L. Braun

**Affiliations:** ^1^Hā Kūpuna National Resource Center for Native Hawaiian Elders, Thompson School of Social Work & Public Health, University of Hawai‘i at Mānoa, Honolulu, HI, United States; ^2^Department of Social Work and Hā Kūpuna National Resource Center for Native Hawaiian Elders, Thompson School of Social Work & Public Health, University of Hawai‘i at Mānoa, Honolulu, HI, United States; ^3^ALU LIKE, Inc., Honolulu, HI, United States; ^4^Office of Public Health Studies, Principal Investigator, Hā Kūpuna National Resource Center for Native Hawaiian Elders, Thompson School of Social Work & Public Health, University of Hawai‘i at Mānoa, Honolulu, HI, United States

**Keywords:** Indigenous, community, traditional healing, advice to practitioners, Hawai‘i

## Abstract

Native Hawaiians are proud and resilient people who have endured significant impacts from colonization. Despite being in a time of vibrant cultural revitalization, Native Hawaiians have a shorter life expectancy than other racial and ethnic groups in Hawai‘i. The primary aim of this paper was to share data from the first year of a 5-year study with Native Hawaiian kūpuna (elders) on their experiences with healthcare, along with barriers to accessing healthcare. Ten kūpuna living in rural areas of Hawai‘i participated in three interviews each, which were held in an informal, talk-story style. The first interview focused on establishing rapport. The second interview focused on the kūpuna's strengths, resiliencies, and what they would like to pass to the next generation. The third interview focused on the elders' experiences with healthcare, which is the focus of this paper. All ten kūpuna reported growing up with limited access to Western healthcare; rather, their families successfully treated many illnesses and injuries with lā‘au lapa‘au (Hawaiian herbal medicine) and other traditional healing practices, as they had done for generations. As Western medicine became more prevalent and accessible, they used both, but many preferred holistic treatments such as prayer, a return to the traditional diet, and lā‘au lapa‘au. As a group, the kūpuna rated their health as fair to good; two had diabetes, two had cardiovascular disease, four had neuropathies, and five were cancer survivors. The kūpuna reported high turnover among providers in rural communities. Limited access to specialists often required them to travel to Honolulu for care, which was costly and especially difficult during coronavirus disease 2019 (COVID-19). Regardless of provider ethnicity, the kūpuna appreciated those who took the time to get to know them as people and respected Hawaiian cultural practices. They advised that Western providers speak honestly and directly, have compassion, and build connections to patients and their communities.

## Introduction

This paper reports on first-year findings from an ongoing, 5-year study conducted with a community partner on strengths, resiliencies, and healthcare experiences among Native Hawaiian kūpuna (elders). Presented first is a short history of the Kānaka Maoli, the Indigenous people of the Hawaiian archipelago. Although the elements of the colonization experience of this group are similar to those of the Indigenous people elsewhere, this review provides readers with a more specific context for the current (poor) health status of Native Hawaiians.

### History

Kānaka Maoli (Native Hawaiians) are the Indigenous people of the Hawaiian archipelago who used their knowledge of the stars, currents, winds, sea life, and birdlife to voyage to the islands by traditional canoe over 1,500 years ago (1). They traveled thousands of miles north from Polynesian islands in the South Pacific, eventually establishing a society in Hawai‘i. Early foreign visitors noted in their ship logs that the Kānaka Maoli were of robust health and had great strength (2).

The early Hawaiians established and evolved a number of healing practices to address physical, mental, emotional, social, and spiritual health conditions. For example, lā‘au lapa‘au includes the gathering, cultivation, preparation, and administration of herbal medicines with pule (prayer) throughout. Many plants have several different uses and health effects depending on factors such as, “where the lā‘au was grown, when it was gathered, how it was gathered, who it was gathered by, and its age” (3). Other traditional healing practices include ho‘ohāpai and ho‘ohānau keiki (induction of pregnancy and baby-delivery), ‘o‘o (simple surgery), hāhā (palpation), ha‘iha‘i iwi (bone-setting), lomilomi (massage), and ho‘oponopono (a process of conflict resolution) (4, 5). In addition to treating physical ailments, resolving spiritual imbalances and social conflicts are important elements of Hawaiian healing (6).

Two important events that brought drastic changes to Hawai‘i were the arrival of Western sailing ships, starting with Captain James Cook in 1778, and the arrival of the first Christian missionaries in 1820. They first brought mass diseases such as tuberculosis, polio, chickenpox, measles, venereal diseases, and influenza. Death from disease and intercultural clashes largely cleared the way for the imposition of a new value and belief system brought by missionaries and settlers from the West that denigrated the Kānaka Maoli's ways of life and led to their economic and political disempowerment (7). Population estimates of the Kānaka Maoli prior to the arrival of Captain Cook in 1778 range from 100,000 to 1.5 million people (8). A little more than 100 years later in 1890, the native population had collapsed to 40,000 people, a 60–97% decline (9).

In 1839, King Kauikeaouli (Kamehameha III) introduced a constitution that created an elected legislative body and codified laws based on Hawaiian customs, while incorporating concepts from Anglo-American common law. This new system ultimately limited the monarch's own power. This was an important decision that made the budding Hawaiian Kingdom more recognizable to global powers as a nation-state, and the Kingdom was formally recognized by France and England in 1843 (10). However, the people struggled for control of their ‘āina (land), rights, and resources as a cash economy took hold in Hawai‘i. In the early 1890s, Queen Lili‘uokalani, at the urging of her people, attempted to regain power that was stripped from the crown by the 1887 “Bayonet Constitution” that her brother, King Kalākaua, was forced to sign under the threat of violence (10). Almost identical to the 1864 constitution, Queen Lili‘uokalani's proposed constitution would have removed or lessened race, language, and land ownership requirements to vote and would have restored the crown's executive powers (10). The day after the Queen attempted to promulgate a new constitution, she was overthrown by a group of white missionary descendants who, in collusion with John L. Stevens, the US Minister of Foreign Affairs, supported the landing of US Marines while they proclaimed themselves the provisional government. Stevens, in turn, used his status as the minister of the US to recognize the self-proclaimed provisional government as legitimate (10). This act violated several treaties held by the US and the Kingdom of Hawai‘i, including the Reciprocity Treaty of 1887 and the Tyler Doctrine of 1842 (11).

Efforts throughout the nineteenth century to keep Hawaiian lands under Hawaiian care were undercut by the overthrow. Prior to the overthrow, foreign settlers could only lease crown and government lands. But once in place as the provisional government, large tracts of previously inalienable lands were sold off to sugar planters, ranchers, and other business interests (12). In 1894, the usurpers proclaimed themselves the Republic of Hawai‘i and continued to advocate that Hawai‘i be annexed by the US. The Kānaka Maoli mounted a massive campaign, resulting in 38,000 signatures on petitions against the annexation that the Queen hand-delivered to Washington DC in 1897 (10). The petitions were successful in that an international treaty of annexation was never ratified. However, in 1898, Hawai‘i was illegally seized by a joint resolution of the US Congress called the Newlands Resolution (10). The ~1.75 million acres of the crown and government lands that were not sold following the overthrow were illegally seized by the US Government (11). Subsequent changes in the political status of Hawai‘i were built upon the false premise of annexation, including the 1900 Organic Act that created the Territory of Hawai‘i and the 1959 Statehood Act (13).

The period after annexation in 1900 and before statehood in 1959 is called the Territorial period. The current cohort of kūpuna (aged 60 and older) were born in this era that was marked by the heavy pressure of American acculturation. World War II, in particular, had a drastic and long-lasting effect on the people and land in Hawai‘i. Today, the US military occupies more than 228,000 acres of land in Hawai‘i for bases, military family housing, warfare training, and bombing practice, and 20% of the island of O‘ahu, the most populous island in the archipelago, is controlled by the US military (14). This era also saw the rise of tourism as the main economic driver in Hawai‘i, which created visibility and representation for the Kānaka Maoli, but also forwarded exploitative and problematic stereotypes, like objectified hula girls and kitschy tiki bars.

At the turn of the twentieth century, plantation workers were brought in from the Philippines, Korea, Spain, Puerto Rico, and other countries, as earlier contract laborers from China and Japan moved on (or were forced to move on) to other places or work (15). People of foreign birth would eventually outnumber Native Hawaiians. Today, the Kānaka Maoli remain an ethnic minority in their homeland, with only 28% of the population identifying as Native Hawaiian alone or in combination with other ethnicities (16).

A significant piece of legislation that aimed to reconnect the Kanaka Maoli to the land from which they had been dispossessed is the Hawaiian Homes Commission Act (HHCA) of 1921. This Act set aside 200,000 acres of land to be leased out in small parcels to eligible “native Hawaiians” for farming, ranching, and housing. For the first time, blood quantum was used to determine one's eligibility for welfare as a “native Hawaiian.” Applicants need to have at least 50% Hawaiian blood quantum to obtain land and are only allowed to pass on parcels to people with 25%. Though many have internalized the concept of blood quantum, the Kānaka Maoli would traditionally prioritize mo'oku‘auhau (genealogy) as a foundation for one's cultural identity. This new definition for “native Hawaiian” created an arbitrary distinction between kānaka based on blood quantum that did not previously exist. HHCA leaders explained that this act was put in place to benefit the native Hawaiians, as they were combating disease, urbanization, and dispossession at the time. However, the introduction of the blood quantum concept ended up racializing the identity of the Kānaka Maoli. Unfortunately, the distribution of these lands has been slow, with many applicants waiting decades for a home. Many of the existing homesteads house multi-generational families and are in sore need of upgrading and repairs (17).

Throughout the different political systems that have governed Hawai‘i over the last two centuries (multiple chiefdoms, kingdom, provisional, republic, territory, and state), different attempts to regulate traditional Hawaiian healing practices have been made. For example, a 1905 Territorial bill outlawed Hawaiian healing with the threat of fines and imprisonment as punishment. Later, healers could apply for a license to practice lā‘au lapa‘au, but to pass the test, they needed to know the Latin names of the different plants accessed for healing. Thus, many kahuna (expert practitioners) and renowned healers who were trained by their elders in ‘Ōlelo Hawai‘i (Hawaiian language) failed the test and were denied licenses (18). Nevertheless, traditional healing practices continued in many families and community settings outside the structure of formal licensure (18).

In the late 1960s, there began a revitalization of Hawaiian culture, language, and traditions. Known as the Hawaiian Renaissance, this era was influenced by anti-war and Civil Rights movements across America and the desire to resist pressures by developers to build expensive housing and hotels for wealthy settlers and visitors in Hawai‘i (19). The events of this time brought about a sea of change in Hawaiian pride and identity that impacted all subsequent generations, including those who are kūpuna today. Amendments made to the Hawai‘i State Constitution in 1978 created the state's Office of Hawaiian Affairs and resulted in some protections for Native Hawaiian traditional and customary practices, including, “guaranteed access of Hawaiians to the mountains and the sea in support of Hawaiian hunting and gathering traditions” (8). Another amendment established ‘Ōlelo Hawai‘i as the official language of the state, along with English. Later, the Native Hawaiian Healthcare Improvement Act of 1988 expanded access to primary care services and traditional Hawaiian healing approaches on each island (20).

The revival of ‘Ōlelo Hawai‘i is particularly exciting. The Kanaka Maoli had a strong oral tradition with a prodigious capacity for creating, memorizing, and transmitting long chants to document the genealogy of their land and its people. Christian missionaries, who began arriving in the 1820s, created a written form of the Hawaiian language as a means to forward their efforts to “civilize” the Kānaka Maoli (10). Reading and writing ‘Ōlelo Hawai‘i was enthusiastically supported by the ali‘i (chiefly class), and by 1834, close to 95% of Native Hawaiians were literate in ‘Ōlelo Hawai‘i (21). More than 100 Hawaiian-language newspapers were published between 1834 and 1948, amounting to over “one million letter-sized pages of typescript text” (22) that are becoming increasingly accessible online. Unfortunately, in 1896, under the self-proclaimed Republic of Hawai‘i, the Hawaiian language was replaced by English as the medium instruction for public schools, effectively banning Hawaiian children from speaking their own language at school (10). The denigration of the Hawaiian language, and later “pidgin,” formally known as Hawai‘i Creole English, continued in the Territorial period as English Standard Schools were created that used proficiency in “proper English” as an admission requirement. After decades of suppression, only 2,000 people were fluent in ‘Ōlelo Hawai‘i in 1983 (23). With the Constitutional changes in 1978, state funds have supported Hawaiian immersion schools (K-12), and local universities now offer advanced degrees in Hawaiian studies and language. Today, an estimated 18,000 people speak Hawaiian, most of them born after 1978 (23). Because of the resurgence of ‘Ōlelo Hawai‘i fluency, more researchers can access the valuable cultural information in the archive of Hawaiian language newspapers, revealing new insights about Hawaiian history, identity, and culture.

### Current Health Status

Although the number of Kānaka Maoli living in Hawai‘i has returned to pre-contact numbers, the population represents only 28% of the overall population today (16). The Native Hawaiian community has won back some rights over the past several decades; however, the population still faces systemic barriers stemming from settler colonialism that affect their physical and mental health. Thus, it is not surprising that Native Hawaiians living in Hawai‘i have a shorter life expectancy, a shorter healthy life expectancy, and an earlier onset of diabetes, obesity, and disability than other racial and ethnic groups in the state (24–26).

Colonization led to militarization, widespread use of pesticides on plantations, uncontrolled development, and racism, and these have contributed to an increased risk of cancer, birth defects, infant mortality, and chronic diseases (such as asthma, diabetes, and cardiovascular disease) in Native Hawaiians (27–29). Many Native Hawaiians have been forced to move farther away from city centers and/or joined with other family members in multigenerational households, as housing options have become increasingly unaffordable. In rural areas, these individuals and families have reduced access to transportation, healthy food choices, quality schools, and safe walking paths. Due to these factors, Native Hawaiians exhibit increased stress levels and are exposed to more violence and crime, which negatively affect their mental and physical health (29–31). While each Indigenous group and culture is unique, the negative effects of colonization on Indigenous health are not; many other Indigenous groups report similarly compromised health as a result of colonization (32–36). This section has described the unique colonization experiences of the Kānaka Maoli that continue to compromise the health status and shape the healthcare experiences of today's kūpuna.

### Purpose

Within the context of this history and given the shorter life expectancy of the Kānaka Maoli, the purpose of this paper is to share preliminary information on the healthcare experiences of Native Hawaiian kūpuna in rural areas, along with barriers to accessing healthcare and kūpuna advice for providers. These data are important to furthering the mission of Hā Kūpuna National Resource Center for Native Hawaiian Elders (Hā Kūpuna) at the University of Hawai‘i at Mānoa, which is to improve Native Hawaiian health by advancing the knowledge and skills of local providers, students studying health and social services, and developers of tailored resources and services for Indigenous elders.

## Materials and Methods

### Study Approach

For this 5-year study, Hā Kūpuna worked closely with the non-profit organization ALU LIKE (a Hawaiian phrase for working together), which is the only provider in Hawai‘i who receives Title VI funding from the U.S. Administration on Community Living (37). Established in 1975, ALU LIKE provides education, vocational, and social programs to different age groups of Native Hawaiians across the state. Their kūpuna program supported by Title VI provides meals, activity, and support to elders through 14 sites across five islands, with programs located in Hawaiian communities and homesteads.

This ongoing partnership between Hā Kūpuna and ALU LIKE is centered in Indigenous research methodologies, which privilege Indigenous ways of knowing and being, emphasize relationships and accountability for all involved in the research project, and seek to benefit Indigenous communities rather than to extract data in ways that reproduce colonial power structures (38–40). As in other Indigenous communities, extractive practices of research have previously caused many Native Hawaiians to distrust research, reporting that researchers tend to gather and publish data that perpetuate negative news about their health and do not use research as a means to improve the educational, economic, social, or health conditions of their communities (41).

To contrast these historical disservices, the partners designed a 5-year study to reflect ALU LIKE's six core values – pono (do the right thing), kuleana (practice responsibility), mālama (be caring), laulima (be cooperative), ho‘omau (be patient, persevere), and ho‘okina (take initiative and forge ahead) (37). A key decision was to interview each kūpuna three times. The main purpose of the first interview was to establish rapport, the second to learn about elders' strengths, resiliencies, and what they would like to pass to the next generation, and the last focused on the elder's experiences with healthcare and social services. This allowed the research team to develop relationships of mutual exchange with the kūpuna. The partners also thought the three-interview series would be well-accepted in early 2021, as ALU LIKE sites were closed due to coronavirus disease 2019 (COVID-19), and the kūpuna had fewer opportunities for socialization. The study was approved by the University of Hawai‘i at Mānoa Institutional Review Board. This report follows the Consolidated Criteria for Reporting Qualitative Research (COREQ) guidelines (42).

### Sample

ALU LIKE site directors on each island were asked to identify three or four kūpuna that used their services who would be willing to participate in a series of three interviews. The ALU LIKE staff took into consideration their prior knowledge of each kūpuna's willingness to talk and share, ability to commit time to three interviews, and whether they thought the kūpuna would find the project fun or interesting. Then, they approached potential participants. At some sites, the staff approached potential participants directly, while at other sites, a written description of the project was shared, and the kūpuna contacted the staff if interested. Participants were solicited first from the island of Moloka‘i (population 7,345), next from the island of Kaua‘i (population 66,921), and next from the Hilo area of the island of Hawai‘i (population 45,056). Our goal was to hear from kūpuna from different islands and to minimize the burden on any one site director, who had to assist the kūpuna with consent forms and/or connecting to the internet for the interviews conducted over Zoom. Because of the close relationship that the ALU LIKE staff have with kūpuna members and their familiarity with each kūpuna's willingness and ability to participate in a project such as this one, no one turned down a direct invitation to be interviewed. Between January and July 2021, interviews were completed with ten kūpuna.

### Measures

The interview questions were designed by both Hā Kūpuna and ALU LIKE. Open-ended questions that guided each of the three interviews are shown in Table 1. The interview questions were pilot tested by the members of the research team, and then revised for clarity. The third interview solicited information on health practices while growing up, about positive, negative, and preferred experiences with healthcare providers and services, caregiving experiences and preferences, and advice for providers. However, these topics also came up in the other interviews, exemplifying how health affects all aspects of life from beginning to end. Relevant data from the first two interviews were considered in the analysis.

**Table 1:** Interview questions.

**Interview 1—Introduction**
1.1 Tell us about what it was like growing up. Where are you from? Include family names, places, and additional background information 1.2 How about today? Where do you live? What is your living situation? 1.3 What is a typical day like for you? 1.4 How are you coping with COVID? 1.5 How long have you been participating with ALU LIKE? What do you like about the ALU LIKE program? 1.6 Can you tell us about a kūpuna that was influential in your life?
**Interview 2—Generational**
2.1 Last time we talked about “name of kūpuna.” What are some things you learned from your kūpuna? What thing do you wish you had learned from your kūpuna? 2.2 What kinds of things do you want to pass on to your mo‘opuna or the next generation? 2.3 Is there anything you would have changed in your life, that can offer lessons to the next generation? 2.4 Tell us about something you've accomplished or are really proud of? 2.5 All life has challenges. What has sustained you through hard times? 2.6 What kinds of things do you love to do and that bring you peace?
**Interview 3—Healthcare services and programs**
3.1 Tell me about what kind of health practices your ‘ohana engaged in when you were growing up. To what extent did your family take care of health/illness at home and to what extent did they consult cultural practitioners or medical professionals? 3.2 Where do you receive healthcare services today? 3.3 Describe a positive experience with a healthcare provider. What made it positive? 3.4 Describe a negative experience with a healthcare provider. What made it negative? How could this experience have been improved? 3.5 What makes you comfortable with a provider? What is important for you in building trust in a provider? 3.6 How should service providers learn more about Hawaiian community and culture? 3.7 What senior programs have you really enjoyed? What about them did you enjoy? 3.8 What programs or services would you like to see more of? 3.9 What have been your experiences with caregiving for your older family members? 3.10 What do you hope for your own care when you get older and might need every-day help? 3.11 What key message would you like health and social service providers to hear?

The kūpuna completed a background datasheet, which was administered by the ALU LIKE staff prior to the interview. Researchers also went through each participant's background datasheet during the final interview to clarify and verify the answers. The datasheet solicited age, gender, education, island residency, if they lived on homestead land, ratings for their health, and access to various forms of healthcare or various types of healthcare providers (from 1 = poor to 4 = excellent). Additional descriptive information was gleaned from the series of interviews, including information on religion, experiences being raised by someone other than one's biological parents (often through the traditional practice of hānai or adoption by another family member), ‘ai pono (healthy eating practices), experiences with lā‘au lapa‘au and other traditional health practices, and specific health status or diagnoses.

### Procedures

The Hā Kūpuna research team consisted of four interviewers (SM, KK, KH, and RB), three of whom were Native Hawaiian; two interviewers were university faculty with graduate degrees, while the other two were graduate students. All interviewers had previous experience with qualitative research. Two interviewers attended each interview. To assure the safety of the kūpuna and interviewers during COVID-19, all interviews were conducted and recorded using Zoom. The participants connected to the Zoom meeting in a private space at an ALU LIKE office or at the kūpuna's home, depending on each participant's preference. If the kūpuna participated on-site at ALU LIKE, then a staff person assisted with technical difficulties whereas if the kūpuna participated from home, then the Hā Kūpuna interviewer would assist them with technical difficulties posed by this interview method.

Each set of three interviews was typically conducted over the course of a month. Before starting to ask questions, the interviewers introduced themselves, explained the full project, and confirmed each elder's consent. The first interview, which usually lasted an hour, proceeded in a talk-story style, allowing the kūpuna to recount stories and reveal lessons in response to questions (43). Interviewers listened and learned, allowing the kūpuna to take the time they needed to tell their stories without interruption, which signifies respect. As the kūpuna shared their family history, the interviewers and the kūpuna also discovered genealogical and community connections, which helped to build trust. The first interview ended with the kūpuna recounting a story about one of their elders, setting the stage for the second interview on intergenerational learnings and teachings. The third interview focused on experiences with healthcare and service providers. The interviews often lasted for 2 h because a good rapport had been established and the kūpuna felt comfortable sharing their stories. The multi-interview process also provided a holistic view of each participant, not just as someone who interacts with healthcare but as someone who has lived a long and full life contributing to their family and community.

Reciprocity is an important part of community-based research and a Hawaiian cultural value. To thank participants for their time and willingness to share, each kūpuna was sent a makana (gift) of māmaki leaves (herbal tea) and pa'akai (salt), along with a flash drive of all three of their interview videos and transcripts. Additionally, the staff prepared a 2-page story about the kūpuna, featuring their key achievements, embraced values, and the lessons they wanted to pass to the next generation. In writing and refining these stories, the researchers continued to interact with kūpuna following the interviews, allowing them to review all materials for accuracy and comfortability.

### Analysis

Video recordings were transcribed using voice-recognition software, then, a team member would go through the entire transcript to fix errors. It took about 4 h to edit 1 h of dialog, which included Hawaiian family and place names, as well as Hawaiian and Pidgin words and phrases, unrecognized by the software. The most fluent Hawaiian speaker on the team then reviewed and further cleaned each transcript.

Thematic analysis (44) was used to analyze the data. This flexible approach allows researchers to systematically analyze qualitative data while outlining their own theoretical assumptions about the data. A multi-step, reiterative process was used to create the codebook. First, a deductive approach was used to generate an initial codebook based on the interview questions. The team also met throughout the interview and transcribing process to discuss and agree on major themes identified in the data from the interviews, and these discussions led to additions to the codebook. The researchers took a semantic approach to the analysis by analyzing the kūpuna's words at face value rather than positing underlying themes beyond their words. They used a realist approach to epistemology, interpreting the kūpuna's words as direct reflections of their lived experience, rather than interpreting their responses as socially constructed reflections.

After the initial codebook creation, the researchers pilot tested the codebook on one interview each and met to refine the codebook based on the themes gleaned from the transcript each person had read. Finally, all interviews were re-coded by two researchers, and their results were compared on an Excel spreadsheet. This spreadsheet was also used to display kūpuna quotes across themes to assist in summarizing the qualitative data.

## Findings

### Participants

The characteristics of the sample are presented in Table 2. Of the 10 participants, four resided in Kaua‘i, four resided in Moloka‘i, and two resided in Hawai‘i island. Four of the 10 respondents lived in Hawaiian Homestead lands, and six did not. Nine (90%) identified as female, and the average age of the sample was 72.8 years old. Seven (70%) had attended college. All had a primary care provider, and all had health insurance through Medicare and supplemental insurance either privately or through Medicaid. None of the kūpuna rated their health as excellent; rather, half the sample rated their health as good and half as fair. While six (60%) rated their access to primary care as excellent, only three (30%) rated their access to specialty care as excellent, and only two rated their access to mental health services as excellent. Two others did not answer the question about access to mental health services at all because of their unfamiliarity with these services.

**Table 2:** Participant characteristics (*n* = 10).

	**Mean (range)**	**Frequency (%)**
Age	72.8 (68–86)	
**Gender**		
– Male		1 (10)
– Female		9 (90)
**Resident**		
– Hawai‘i		2 (20)
– Kaua‘i		4 (40)
– Moloka‘i		4 (40)
**Homestead**		
– Yes		4 (40)
**Highest education**		
– High school		2 (20)
– College		7 (70)
– Graduate		1 (10)
**Health insurance**		
– Yes		10 (100)
**Established primary care provider**		
– Yes		10 (100)
**Self-Rated health (1** **=** **Poor**, **4** **=** **Excellent)**	2.5 (2–3)	
– Poor		
– Fair		5 (50)
– Good		5 (50)
– Excellent		
**Rate access to primary care** **(1** **=** **Poor, 4** **=** **Excellent)**	3.4 (2–4)	
– Poor		
– Fair		2 (20)
– Good		2 (20)
– Excellent		6 (60)
**Rate access to specialty care** **(1** **=** **Poor, 4** **=** **Excellent)**	2.8 (1–4)	
– Poor		1 (10)
– Fair		3 (30)
– Good		3 (30)
– Excellent		3 (30)
**Rate access to mental health services (1** **=** **Poor, 4** **=** **Excellent)**	2.6 (1–4)	
– Poor		2 (20)
– Fair		1 (10)
– Good		3 (30)
– Excellent		2 (20)
– Did not answer		2 (20)

Through the course of the conversation, participants disclosed information about their careers. Four (40%) had worked in healthcare (nurse, nurse's aide, patient advocate, administrator), two (20%) in social services, two in transportation, one in education, and one in entertainment. In terms of religious practices, nine (90%) recounted regularly attending church during their childhood, including affiliations with the Catholic Church, the Church of Jesus Christ of Latter-Day Saints, Missionary Baptist Church, and churches with large Hawaiian congregations. Of these, seven participants were still attending church, and one participant followed Native American spiritual practices. Most of the participants that reported attending church also reported using pule (prayer) in their life as a Hawaiian health practice.

Unlike in many cultures, “in deep Hawaiian tradition, the first-born (hiapo) girl was dedicated to the maternal elders, the first-born boy to the paternal ones (45).” It was still common in the mid-twentieth century, when our participants were growing up, for a child to be hānai or adopted (literally, “fed”) by one's grandparents, aunties or uncles, or even older siblings. Four of the participants in this study were hānai as children and were, at least temporarily, raised by someone other than their parents. It is important to note that there was neither secrecy nor shame associated with hānai; participants framed the experience as being wanted and loved by the broader family, rather than being unwanted by one's biological parents.

### Themes

Qualitative findings were organized by major themes that aligned with the interview questions used to guide the third interview. These included: (1) experiences with healthcare while growing up; (2) positive healthcare experiences; (3) negative healthcare experiences; (4) experiences with social services; (5) caregiving and long-term care; (6) advice for providers.

#### Experiences With Healthcare While Growing Up

Seven of the participants explicitly stated that they did not see a Western provider growing up unless it was for immunizations or a serious health issue, such as a broken leg or a cut needing stitches. One said, “*The only kind of medical thing we had was to go to [hospital] whenever we needed shots.”* Another stated, “*Back then, I guess… you don't go to the doctor because I guess maybe insurance or whatever… I don't know if they had or was expensive… Got to be really serious before you go to the hospital.”* Another explained, “*We never had any cars, we only had horses, no chariots whatsoever. So, we never went to any … medical facility or medical doctor, the Western way. [It] was all the Hawaiian way, the cultural way*.

Eight of the ten kūpuna recounted how their families used lā‘au lapa‘au. One participant remembered:

*If any of us got sick, we had the lā‘au, no other medicine. [M]y grandfather was the one that was a kahuna [expert]… He had all the plants; the lā‘au that he needed if anybody got sick. [T]hey were raised in Waikapu in Waihe‘e where they had the mountains, with everything that they needed, with the lā‘au and the water that came down in their area in their village, so they were raised the same way with using what they had around*.

The herbal remedies mentioned were noni (Morinda citrifolia) or popolo (Solanum americanum) for colds, aloe vera for burns and scratches (liquid or solid form), coconut water for a strong immune system or kidney problems, guava shoots for stomach aches, ‘ōlena (Curcuma domestica) for earaches, māmaki tea (Pipturus albidus) for internal disorders, ti leaf (Cordyline terminalis or C. fruticosa) for fevers, lantana and eucalyptus leaves for congestion, and honohono grass (Commelina diffusa) for cuts. A kūpuna explained:

*When you got burns, it wasn't run to the hospital…my tutu man [grandfather] would go outside or my father would go outside, get aloe for the burns… put it on the burns… I remember one time I was in church… My son had a really high fever and they went and got ti leaf. One of the kūpunas got ti leaf and put it on him to bring the fever down. So, it wasn't… run to the hospital, run to the doctor, it was always natural stuff*.

Faith and prayer were also important in the practice of lā‘au lapa‘au, as explained by this participant:

“*When I tell them [grandchildren] take the lā‘au, I always said, ‘You have faith and prayer. You have to verbalize. I can say it, but you need to know because it's you, your body.' And if they don't want to then I said it's useless for me to give to you.”*

Another participant remembered going with her mother to harvest the plants:

*The thing I remember about my mom, she said to be quiet. And she'd go and stand, and she would be standing there and I'm looking at her and she's offering a prayer of which one she's going to pick up… She would pick up the ones that was the most healthy. And she picked up enough to last—she would say five kāunas, meaning five days she was going to be giving me morning and evening, morning and evening. So that medicine, the lā‘au that she picked up, lasts… It was always in the early morning before everything started, yeah?… And I would see her wrap it up in ti leaf…and she would bring it home and prepare it. And she would set it aside. And we had this bamboo safe…covered with cloth so that the flies don't get in there. And she would put it in that because we didn't have refrigeration, yeah? Well, she would put it in there. And that was a preparation of the lā‘au. Then when I was given it, she would go out and burn it over the fire. She'd build a small fire, and she would…just kind of steam it there because the ti leaf would be the one to give it substance…And she would tell me that you must believe that it's going to heal you. And so she would say that prayer before she would give it to me*.

Four participants mentioned using lomilomi, a Hawaiian massage practice, to treat aches and pains, as well as huli stomach (colic or other stomach pain, especially in babies) (6). A participant said, “*Gosh…, I don't go the doctor for huli stomach. When my kids had, I take them to a lady and she fixed it right away… And she worked on him with oil, heated up the oil, and lomilomi the stomach.” Four* kūpuna talked about the family's use of ho‘opono‘pono to address conflicts and increase harmony. A kūpuna likened it to “*family counseling… you get together and talk about it… If there's some kind of hakakā [strife or argument]…it's good healing… you're not going around with this… that you're mad at somebody.”* Another Hawaiian health practice, mentioned by five participants, was ‘ai pono, defined as healthy eating. A participant summarized the healthy diets of their youth by saying:

*We ate healthy… we didn't have junk food…When we stayed with our tutu man [grandfather] and tutu lady [grandmother], there was always a bowl of poi … And… growing up Hawaiian, that was our healthy way of eating. Until what? We were like Westernized and even that my father fought it… But for us, the next generation, it was easier to go Western, McDonald's, and stuff like that*.

As Western medicine became more available over the years, participants incorporated practices from both modalities. All the participants reported having access to and utilizing a primary care provider for basic health needs. Many participants also needed specialists for specific conditions such as cancer, diabetes, kidney problems, spinal issues, neuropathy, and heart problems. However, many still preferred natural products and treatments, as noted by this participant:

*Sometimes when they prescribe medication and all that? Sometimes we think, “Well, let's just go the Hawaiian way…” Medicine or like, tea or whatever. I mean, I'm not going to tell the doctor that, but… if they do that to me, it's like I'll just try this way… the way I believe is going help me*.

#### Positive Healthcare Experience

All ten kūpuna currently used Western medicine in combination with Hawaiian approaches, and all ten recounted positive experiences with a primary care doctor or a specialist. One participant mentioned, “*I like him [the doctor], he's so pleasant… he relaxes me and so does his staff… He likes to ask you about the family and how things are going.”* Several others talked about positive experiences with physicians that took the time to explain to them what was going on with their health. A kūpuna diagnosed with pneumonia said:

*She showed [the X-ray] to me and it was all cloudy, and I said “Oh, no wonder I had a hard time breathing”… And so, I was pleased because she went the extra step… to have me do an X-ray instead of just sending me home with cough medicine… She was really thorough*.

Another said: “*I liked him because he told me the straight facts to me… to my face, what was wrong with me… And so compassionate… That's the kind doctor that you feel comfortable with.”* Another had a good experience because of the increased accessibility to her physician through a patient portal.

*He'd tell me what was happening and what was going and why… The same things he told me in the office was written down in black and white, so that I could follow… I can say that that was the best doctor I had. Because he told me things that I could do when I go home. And then, if I had questions I will put it into my patient portal. So even though I'm not there, he just gives updates of what can be done, how I'm doing*.

Five kūpuna noted receiving care from one of the growing numbers of Native Hawaiian physicians in the state. For example, one remembers “*Sitting in the office and all of a sudden comes this nice-looking, Hawaiian guy. He's kind of on the big, hefty side, right? But he's smiling… he has this big smile on him and I'm thinking to myself, “Oh, what a wonderful doctor. He's always smiling.”* Another kūpuna had a positive experience with the Native Hawaiian oncologist who treated her advanced cancer. She was especially impressed because this physician was from the same neighbor island and graduated from the same high school that she did.

*[He] happened to be alumni like myself, and he just looked me as close as I am to you and he said, “Do you trust me? We don't have time.” And my thought was “Wow, I must be in stage four or something.” I never asked. I never wanted to know, even if he wanted to tell me… So I put all my trust in [that doctor]*.

#### Negative Healthcare Experiences

When asked about negative experiences, four participants gave examples of physicians with poor communication styles. For example, a kūpuna shared:

*When I was going through my cancer, my breast cancer, I did not like her at all… I thought she was fake. She was never in that room when they were doing the radiation with me. But I would get dressed, go in a room, and she'd come in and hop herself on the chair and say, “well, how do you feel?”… When I asked her a direct question, I said, “What are you seeing when you guys take X-rays every day?”… [she says] “Well, you know…” And I said, “No, I don't know. That's why I'm asking you.”*

Participants primarily complained about high turnover in providers, lack of access to specialty care on their islands, and discomfort with traveling to Honolulu for specialty care. Of the six kūpuna that shared stories about the high turnover in providers, one said, “*I used to have a man doctor, and then he left. And after he left, I didn't have anybody.”* Another said of a popular physician at a community clinic, “*[He] knew a lot of patients. When it was time for him to leave…we were so sad because he was such a great doctor.”* Another stated, “*You get comfortable too with the doctor. And then he's gone, he's gone. Poof, gone.”* Another noted:

*When I went one time, they said she was leaving…[and] I cried. I went “Oh my gosh, this big lady [meaning herself] crying.” But she was so nice… I really could open up to her and talk to her… Here on [our island], there is such high turnover in medical staff and doctors and nurses. They recruit nurses and young doctors from the mainland America… maybe one or two years' experience…in completely different frameworks. And they come here because it's a chance to gain experience. It's a chance to get in the ocean… But then the wages are low comparatively. Their debt is high*.

Eight participants commented on the general lack of access to specialists and the short time visiting physicians had to spend with them. For example, one participant stated, “*Why do we have to go to Honolulu to see specialists? Why don't the specialists come here? Huh? We got plenty cancer people here, what happened to our treatment?!”* However, when visiting physicians came to their islands, patients often had to wait a long time to see them. For example, an elder explained:

*There was this one doctor… a specialist right, that used to come [to our island] and see my mom. The appointment is at 11 o'clock and it's already 11:15, it's turning 12, and it's turning 12:30 and 1:00. And I'm blowing already. I mean talk about a volcano… So my cousin is hearing me talking…and he's sitting next to me and he said, “Hey, it's nothing new. Wait. Maybe she gon walk in at four o'clock.”*

Another elder had worked as a patient advocate and noted that visiting providers prioritized other tasks over scheduled patient appointments. She recalls advocating for patients, by saying to a visiting specialist: “*If you have other things, can you just make it so you can do it when you no longer have patients? Maybe during your lunch hour, you can be talking and eating at the same time, instead of having people waiting. There are patients out there.”*

Limited specialty healthcare on neighbor islands often required the participants to travel to Honolulu for care, which can be costly, dangerous for kūpuna to go alone, and was especially difficult to arrange during COVID-19. One participant lamented that the sole airline that services their island, lacks adequate equipment, like a ramp, to assist people with limited mobility to board the aircraft. “*How can you go – somebody with a wheelchair or somebody that cannot walk – put them on [a] forklift?”* Concerning COVID-19, one participant stated,

*I was good until the COVID started and then my doctors would call to schedule my appointment in Honolulu. And I'd say, “Oh, I don't want to go…” Because I refuse to go, I'm scared. I'd rather stay home. I was supposed to be taken off a medication and, because I am not there physically, I still have to take it now. I was supposed to be off since last year in November but I'm still taking it*.

Even with the access to care on O‘ahu, two kūpuna talked about feeling unseen. One participant stated, “*Honolulu is huge… I get frustrated living in Honolulu [where] I'm just a number, just doing my vitals, boom, seen one minute, boom, I'm out… I think it's very, very important to have [a deeper] relationship with your doctor.”* Another was not happy to have to see physicians through telehealth. For example, a participant stated, “*How can you, on the opposite side as the doctor, see me through Zoom and say, ‘Oh… like this and like that.' Excuse me?! Can you – you can only see the top. Can you see my bottom [half]?”*

#### Experiences With Social Services

The primary social service that the participants were familiar with was the ALU LIKE program, and the comments were overwhelmingly positive. Although congregate programming was closed during the COVID pandemic, elders shared things they appreciated about the program before the pandemic. An elder explained, “*We learned a lot about aging and about Kūpuna Power, about kūpuna help, and to make us aware of others and their needs and talents.”* Another said:

*ALU LIKE…would have presenters…And it's so interesting because you get one…for diabetes, you have all the cancer programs, and all where you can seek help… Mālama Nā ‘Oiwi [community clinic]…and then your insurances…Your VA for hearing aids. So, you just have to tap your resources and pay attention*.

Another missed the annual health fair, remembering, “*Once a year they have where it's at the stadium where it's for everybody, anybody, all kind of different programs…they give you brochures on whatever services they're giving out…they have lucky number…bingo and food.”*

Another elder appreciated the exposure through ALU LIKE to Hawaiian history, language, and cultural practices, saying, “*We do lauhala [weaving with plant fibers], we study King Kamehameha, Kalākaua… all the old ancient kind of stuff. We dance hula, we play ‘ukulele, we do Hawaiian instruments.”* Another enjoyed the speakers on Hawaiian healing practices, “*In ALU LIKE, they would have people come in… do lomilomi [Hawaiian massage] on us. Oh man, that was heavenly, free kine. Or come in and talk to us about health and stuff like that. And I liked that. I really did.”*

Another remembered interacting with children at a Hawaiian-language immersion school and marveling that they knew more Hawaiian language than the kūpuna, who grew up in a time when the speaking of Hawaiian was discouraged. “*I'm thinking to myself, ‘Oh thank God these kids know it. I don't know what is what.' So…and it's the little ones that are helping the elderly ones. I mean, some of the elderly understand, but me, I have no idea.”*

Another missed the field trips, remembering, “*We even go riding around town or go to the east end and do… just riding! Or we go visit somebody from that end, and we have a picnic, or we go to the beach, or we even go pick seaweed at the beach…We even travel outer island! We went Maui and we went Kaua‘i.”* Another kūpuna gave an example of how the ALU LIKE staff helped her on and off the van, “*Through ALU LIKE…they took care of the kūpunas… stepping up into the van, they always had a stool getting us in, holding onto us coming in or out. I mean they're very, very patient… and you knew that they liked their job.”*

Four gave examples of how ALU LIKE engaged them to teach or help others. A participant told us she was regularly asked to talk about how to trace one's genealogy, and another noted that her husband taught card games like hanafuda (a popular game of Japanese origin played on the plantations). Another said their club used to volunteer at the food bank. Another said, “*We teach the kids at high school. Anybody who invites us, we go to the high schools, to the dropout schools, to the home schools. I mean…even the young children come to us, the Kamehameha children. We do all kinds [of things with them].”*

All ten missed ALU LIKE's congregate programs. A participant said, “*That's what I miss most – being with everybody. I mean, I had something to look forward to. I mean… you would be in this slump if you didn't have friends.”* However, the ALU LIKE staff continued to work for clients. Two noted that the staff organized food delivery to their homes during the COVID pandemic, along with helping them access the food bank. “*There's baskets of – I said baskets now! I mean, some of them come with poi, they get vegetables, they have fish, they have deer meat, they have all kines for our house. The food bank comes! I mean the community they are so helpful.”* Another appreciated that ALU LIKE had provided her a computer, saying, “*I don't feel isolated because through the ALU LIKE program I have a computer at home…I got that through ALU LIKE, so yeah, they trying to meet the needs of the elderly, so that they can stay connected with grandchildren and children.”*

#### Caregiving and Long-Term Care

Eight out of the ten participants mentioned directly caring for a parent, another family member, or a non-related elder at the end of life. For the most part, death occurred at home, rather than at a facility. As a participant explained about her mother, “*She didn't want to be locked in a hospital. So, with the four daughters that she had, we all took turns, watching my mom… I mean at home…all of us… And I worked, everybody worked.”* Another shared about her mother, “*We kind of took turns taking care of her. But when she was coming close [to the end] she said, ‘You know, I'm tired already'…so we got a [hospital]bed for her. And she wanted to stay outside in the patio. She didn't want to be in the house.”* Another participant took care of her brother who had lung cancer, saying, “*I was there to help him, and he had a tracheotomy. He had a tube in him all the time. So whenever he wanted something, I got a bell for him to ring, so that I could be there.”* Another cared for her adult son until he died:

*One day when I was at the house…he was really bad already. He had to use the bathroom…I picked him off the couch. He was already just skin and bones…And when he got done…and I laid him back on the couch, he said, “Thanks, Mom.” I said, “I love you son.” He said, “I love you too.” And the next day he passed*.

Another participant's family found hospice to be helpful, saying:

*Hospice would come and help us… and give us a chance to rest…I thought hospice nurses was one of a kind… I would have a hard time because they get so close to you. They treat the patient like that's their mom… their grandma*.

When asked about preferences for their own care, three felt they could rely on their children to care for them, and seven said they did not want to burden their children. Eight recounted conversations about their future care with their children. A participant shared her conversation with her son:

*My son said, “Mom I'll take care of you,” I said “No, I don't want you to take care of me. I don't want you because you have your family. I'll just be a burden on you… just take me to an…old home care place where they have people to take care of you.” And he said, “No, not going to happen.” I said, “Well that's the way it is. You're not going to take care of me…I have it written over here.”*

Another participant said, “*Just because I don't want to burden my kids…I applied, and I've been paying for long-term care [insurance] for a long, long time.”* Another shared that she had purchased a funeral plan to save her children the worry and expense. Nine of the participants talked about services they knew about that could help kūpuna age in place, the importance of documenting their wishes, and/or the services their private or public insurance plans might cover. The kūpuna mentioned learning about some services, like hospice, through the care of their own family members. It is likely that they also learned about services and advanced care planning through educational programs provided by ALU LIKE.

#### Advice for Providers

There were four main lines of advice provided by participants. First, the elders appreciated the providers who took the time to talk-story and get to know them as people and community members. The kūpuna also enjoyed when the providers shared information about themselves. One participant talked about a physician who visited her in the hospital, “*He come like two, three o'clock in the morning and he'd stay to talk story with you, so I liked him. He was just very, very pleasant.”* Another said:

*Be more caring, be more involved with your patients' lives. I guess you would say, be mindful of what you say. I mean… you have their record right in front of your face, I mean you have the health issues. But what's the underlying? [Some just say] “You have to do this; you have to do that.” Now do they really do it? Coming from my point of view, I don't think so. Because if I want to listen to you, I'll listen to you. But they have to find a different way of trying to communicate with their patients. “Cause some people just turn me off when they talk.”*

The second area of advice was to learn about and acknowledge Hawaiians' experiences with colonization and Hawaiian health practices. A participant felt this would help providers look at and treat patients more holistically, saying:

*You learn and treat the whole person, not just a symptom. And you got to understand where the genesis of that system comes from and treat that instead of just giving a drug to numb… you got to treat the root of it. And they don't …teach that much in medical schools*.

Another participant recommended incorporating both Hawaiian and Western practices in providers' offices, explaining:

*I think I would like to see Hawaiian medicine, along with regular American medicine. I think that would really help in introducing Hawaiian medicine. Because it works! It's just getting it and doing it… and taking it. But it works. I've seen it work*.

The third piece of advice was to communicate directly. Four kūpuna told positive stories about providers who were straightforward when talking about diagnoses or recommendations. One participant declared, “*I liked him [doctor] because he told me the straight facts to me… to my face, what was wrong with me.”* Even if the participant was not on the same page as the provider, they enjoyed it when the provider was willing to take the time to thoroughly explain the health issue or concern so that the patient could fully understand the situation. One participant recounted how a physician drew pictures to help clearly communicate a patient's condition and the necessary care the patient would need to do upon discharge. “*The exam table… it's paper. So, he goes and he draws the big picture, ‘This is this, and this is what's happening, and you need to do this…' So that explanation comes from that doctor or that nurse that will help you to remember what they have to do.”*

The last piece of advice offered was to love your work. Participants found it obvious when their provider did not enjoy the job, which led them to have a negative experience. One participant who had a career in healthcare said, “*Anybody that gets into healthcare, they gotta love it… Because otherwise they be going to work and say ‘Ugh. I wish I wasn't here.' I never had that. I looked forward to going to work.”* Another participant who worked in healthcare advised providers to have compassion for their colleagues and staff, as this helped create a workplace that was more positive for the patient, as well as employees, explaining: “*Take the important things that you can make a difference for patients… keeping up the morale for your workers… Believe in those people, reward them if you can, in some way.”* When healthcare teams and departments have strong relationships internally, participants found that they were treated better and had a positive experience.

## Discussion

There were several key findings from this study. First, most of these kūpuna transitioned from predominantly relying on sophisticated traditional Hawaiian healthcare practices in their early life to today, when they use both Hawaiian and Western practices and would like to see more integration of these approaches. Second, most of the participants had experienced cancer or other serious chronic diseases in their adulthood that required them to interact with specialists to whom they generally had limited access. Third, these Native Hawaiian participants exemplified resilience despite experiencing health problems and limited access to specialty and mental health services in rural communities. Finally, kūpuna valued connection with their healthcare providers, which was established through mutual sharing of and respect for each other's lives. These findings validate the existing literature on healthcare experiences of minority groups and rural residents in the U.S.

Despite growing up in a time when being Hawaiian was considered a liability, the kūpuna had good experiences with Hawaiian health practices. Although Western medicine is appreciated and necessary in managing chronic conditions experienced by the kūpuna, they preferred a more holistic approach to treatment that included lomilomi, lā‘au lapa‘au, and prayer. Similar results have been found in other Indigenous communities, such as the Confederated Tribes of the Umatilla Indian Reservation for which health encompasses environmental, physical, mental, spiritual, and social wellness (46).

In Hawai‘i, positive attitudes toward Hawaiian health practices continue today, as can be seen in other findings that report a high prevalence of co-utilization of Western and Hawaiian practices among clinic patients. In a 2007 study, participants who used both approaches noted that traditional healers took the time to listen and explain diagnoses and treatments, and thus they felt more connected to healers than to physicians (47). With the Native Hawaiian Healthcare Improvement Act of 1988, more clinics have been offering Hawaiian healing practices, especially lomilomi. At least one Federally Qualified Health Center employs a practitioner of both lomilomi and lā‘au lapa‘au who sees patients alongside a Western provider and prescribes herbal remedies that are taken concurrently (or sometimes instead of) Western medicine (48).

Health statistics demonstrate lower life expectancy and high prevalence of a chronic disease among not only Native Hawaiians (25), but in American Indian and Alaska Native communities as well (49). Although participants were not asked to report their chronic diseases, they spoke about having diabetes, heart disease, hypertension, and neuropathy. Half of the participants reported having had cancer, which compares to a lifetime risk of being diagnosed with cancer in the US of about 40% (50). These conditions required them to interact with specialists. While six of the ten kūpuna said they had excellent access to primary care, only three rated their access to specialty care as excellent, and most gave examples of lack of specialists, long wait times for visiting specialists, and impersonal care when they traveled to Honolulu. An abundance of research confirms higher disease prevalence among adults living in rural areas of the US, in part, due to limited access to specialty care and delays in receiving treatment (51). The same study notes a scarcity of mental health services in rural areas, and this was confirmed by our participants, with two stating they did not know enough about mental health services to rate their access to them. Many other Indigenous populations also experience limited health coverage, access to the Internet, and transportation, and these barriers lead to delayed access to care which can lead to more severe illness with fewer options for treatment (49).

Third, participants exemplified resilience despite experiencing health problems and limited access to specialty and mental health services in rural communities. They expressed appreciation for the services they did have, noting the expansion of Western care options in their communities since they were children. They were happy to use health and social services, many of which they learned about through their careers and programs like ALU LIKE. They were especially excited about seeing more options for families, and especially children, to learn the Hawaiian language and culture. Research shows that building and maintaining a positive cultural identity can promote resilience and self-esteem and provide a buffer against stress (52).

Finally, the kūpuna valued connection. The participants gave examples of how physicians had connected with them, and these physicians were the ones that were trusted and whose advice was followed. Connections were built by sharing things about each other's lives and getting to know each other as people within relationships, families, and communities. Research shows that building provider-patient connections is important for patients of all races and ethnic groups. A 2020 study that synthesized data from the literature, primary care encounters, and providers, patients, chaplains, and social workers resulted in five recommendations for physicians as they work with patients: “prepare with intention (take a moment to prepare and focus before greeting a patient); listen intently and completely (sit down, lean forward, avoid interruptions); agree on what matters most (find out what the patient cares about and incorporate these priorities into the visit agenda); connect with the patient's story (consider life circumstances that influence the patient's health; acknowledge positive efforts; celebrate successes); and explore emotional cues (notice, name, and validate the patient's emotions)” (53). Training in these five points should be included in all health professional education programs.

Increased access to Native Hawaiian practitioners may increase the ability of the kūpuna to make connections, as providers with similar cultural backgrounds may have an intuitive understanding of their patients' contexts, worldviews, and values through their own lived experiences. Evidence from a secondary analysis of data from the 2010–2016 Medical Expenditure Panel Survey, suggests that patient-provider racial “concordance contributes to a more effective therapeutic relationship and improved healthcare” (54). Expanding the pool of Native Hawaiian practitioners should also support the integration of Western and Hawaiian healing practices. As part of the Native Hawaiian Health Improvement Act, full scholarships are available to Native Hawaiians pursuing medicine, nursing, social work, psychology, and other health disciplines. Scholarship recipients are required to reciprocate this financial support by practicing in predominately rural Native Hawaiian communities for 2–4 years post-graduation. Data from 2020 suggest that 94% of these graduates have remained employed in their discipline in Hawai‘i and continue to serve Native Hawaiian communities (49).

Connections were also made between the participants and researchers in the course of this study. These connections were facilitated through the disclosing of family names and places, through which participants and researchers discovered shared ancestry, family, and friends. This served to increase the comfort of participants with the interview and resulted in the sharing of more information and stories, as demonstrated by the increasing length of time spent in the third interview compared to the first interview. Establishing these connections helped build rapport, which is especially important in qualitative research on healthcare. Researchers were careful not to express judgment about people they knew in common or about the participants' experiences, but to continue to facilitate the interview through the expression of curiosity and interest (55).

The findings of this qualitative study support several recommendations. First, health resources need to be expanded to increase access to care in rural Native Hawaiian communities. Second, health centers and providers should support the offering of Native Hawaiian health practices alongside Western practices. Third, more Native Hawaiians should be encouraged to enter the health professions, as they should have an easier time establishing rapport with Native Hawaiian patients and are more likely to stay in Hawai‘i to serve Native Hawaiian communities. Finally, training programs for future health practitioners of all racial and ethnic groups should continue to teach students the importance of making connections with their patients by learning about and showing respect for their cultures, contexts, and statuses within their communities.

There were several limitations to the study. First, three interviews were conducted with each elder to build trust, to gain a broad perspective on each elder's life and strengths, and to decrease isolation during a year when congregate programs were closed due to the COVID-19 pandemic. While this approach had many advantages, it limited who was interviewed. For example, although 30 interviews were conducted, information was collected from only ten kūpuna on three of the seven inhabited Hawaiian Islands, limiting the generalizability of findings. Of the ten participants, nine identified as female and one identified as male, which reflects ALU LIKE's service population, which is about 86% female. However, future interviews will strive to include more men in the sample.

Another limitation to this study was that the participants may have been more outgoing and tech-savvy than non-participants as they needed to respond to the call for volunteers, commit to a three-interview series, and be willing to be interviewed using Zoom. Also, given the recruitment strategy, more socially isolated or vulnerable elders may not have been adequately represented and may have reported different experiences than the participants that were interviewed. However, word-of-mouth sharing by the first ten participants about their positive experiences has led other ALU LIKE members to ask to be involved.

The results of this study were shared with the ALU LUKE staff for feedback. Personal stories and transcripts have been provided to kūpuna, and a summary of findings will be presented in the future. However, member checking was not conducted for this current manuscript due to time and resource constraints. This is a limitation of the study in that results reflect researchers' interpretations of participants' responses without further input from the respondents themselves. Social desirability bias was also likely in response to our health services self-rated questions, as many who rated access to services as good or excellent also shared stories of limited access and barriers.

As this project continues over the next 4 years, researchers hope to gain more knowledge about the perspectives of Kānaka Maoli kūpuna on their life experiences, strengths, and resiliencies, as well as their healthcare experiences. These findings will be shared with health and social service students and providers to further the goals of improving access to care and extending longevity for Native Hawaiians.

## Data Availability Statement

The original contributions presented in the study are included in the article/supplementary materials. Further inquiries can be directed to the corresponding author/s.

## Ethics Statement

The study, which involved human participants, was reviewed and approved by UH IRB. The patients/participants provided their written informed consent to participate in this study.

## Author Contributions

KB, SM, and LT conceived the study. KK, KH, SM, and RB conducted the interviews. KK and KB led the writing. All authors participated in data analysis and interpretation. All authors edited and approved the manuscript.

## Funding

This project was supported, in part, by the U.S. Administration on Community Living Department of Health and Human Services, Washington, DC 20201 to Hā Kūpuna National Resource Center for Native Hawaiian Elders (90OIRC0001). Grantees undertaking projects under government sponsorship are encouraged to express freely their findings and conclusions. Support also was received from the Barbara Cox Anthony Endowment at the University of Hawai‘i at Mānoa.

## Author Disclaimer

Points of view or opinions do not, therefore, necessarily represent official government policy.

## Conflict of Interest

The authors declare that the research was conducted in the absence of any commercial or financial relationships that could be construed as a potential conflict of interest.

## Publisher's Note

All claims expressed in this article are solely those of the authors and do not necessarily represent those of their affiliated organizations, or those of the publisher, the editors and the reviewers. Any product that may be evaluated in this article, or claim that may be made by its manufacturer, is not guaranteed or endorsed by the publisher.
